# Assessment of Intramuscular Fat and Correlation with Body Composition in Patients with Rheumatoid Arthritis and Spondyloarthritis: A Pilot Study

**DOI:** 10.3390/nu13124533

**Published:** 2021-12-17

**Authors:** Marc Villedon de Naide, Bruno Pereira, Daniel Courteix, Frederic Dutheil, Lucie Cassagnes, Yves Boirie, Martin Soubrier, Anne Tournadre

**Affiliations:** 1 Rheumatology Department, CHU Clermont-Ferrand, 63000 Clermont-Ferrand, France; vdnmarc@hotmail.fr (M.V.d.N.); msoubrier@chu-clermontferrand.fr (M.S.); 2 Biostatistics Unit (DRCI), CHU Clermont-Ferrand, 63000 Clermont-Ferrand, France; bpereira@chu-clermontferrand.fr; 3 Laboratory of the Metabolic Adaptations to Exercise under Physiological and Pathological Conditions (AME2P-EA 3533), University Clermont Auvergne, 63000 Clermont-Ferrand, France; Daniel.courteix@uca.fr; 4 Preventive and Occupational Medicine, CHU Clermont-Ferrand, 63000 Clermont-Ferrand, France; fdutheil@chu-clermontferrand.fr; 5 Service de Radiologie Adultes, CHU Clermont-Ferrand, 63000 Clermont-Ferrand, France; lcassagnes@chu-clermontferrand.fr; 6 Thérapies Guidées par l’Image, Institut Pascal, UMR 6602 CNRS-SIGMA-Université Clermont Auvergne, 63000 Clermont-Ferrand, France; 7 Service de Nutrition Clinique, CHU Gabriel Montpied, 63003 Clermont-Ferrand, France; yboirie@chu-clermontferrand.fr; 8 Unité de Nutrition Humaine, UMR 1019 INRAe-Université Clermont Auvergne, 63000 Clermont-Ferrand, France

**Keywords:** rheumatoid arthritis, spondyloarthritis, body composition, muscle, fat

## Abstract

Rheumatoid arthritis (RA) and spondyloarthritis (SpA) are associated with changes in body composition. Ectopic intramuscular fat (IMAT) may alter muscle function and contribute to cardiometabolic disorders. In a pilot study, we analyzed IMAT in the calf with peripheral quantitative computed tomography (pQCT) and examined correlations between IMAT quantity and body composition parameters. In 20 patients with active RA and 23 with active SpA, IMAT was correlated with visceral fat (VAT; r = 0.5143 and 0.6314, respectively; *p* < 0.05) and total lean mass (r = 0.5414 and 0.8132, respectively; *p* < 0.05), but not with whole body fat mass. Total lean mass mediated 16% and 33% of the effects of VAT on IMAT in RA and SpA, respectively. In both RA and SpA, calf muscle area was correlated with total lean mass (r = 0.5940 and r = 0.8597, respectively; *p* < 0.05) and fat area was correlated with total body fat (r = 0.6767 and 0.5089, respectively; *p* < 0.05) and subcutaneous fat (r = 0.6526 and 0.5524, respectively; *p* < 0.05). Fat area was inversely correlated with handgrip and walking tests, and it was associated with disease activity and disability. We showed that ectopic IMAT, measured with pQCT, was correlated with VAT, but not with total body fat, in RA and SpA. This result suggests that metabolically active fat was specifically associated with IMAT.

## 1. Introduction

Rheumatoid arthritis (RA) and spondyloarthritis (SpA) are associated with increased cardiovascular risk [1]. These associations are partly due to classic cardiovascular risk factors, but they are also due to the presence of systemic inflammation which leads to metabolic disorders, such as insulin resistance and altered body composition. Studies have shown that sarcopenia was 2- to 3-fold more frequent in patients with RA than in healthy controls [2,3,4,5], and sarcopenia was associated with ectopic fat deposits [6]. Moreover, cachexia-associated metabolic disorder might explain the finding that a low body mass index (BMI) was associated with an increase in cardiovascular mortality by 2- to 3-fold, in patients with active RA compared to the general population. Despite conflicting results, in SpA, the prevalence of sarcopenia was also correlated with disease activity and severity [7,8,9].

**Figure 1:**
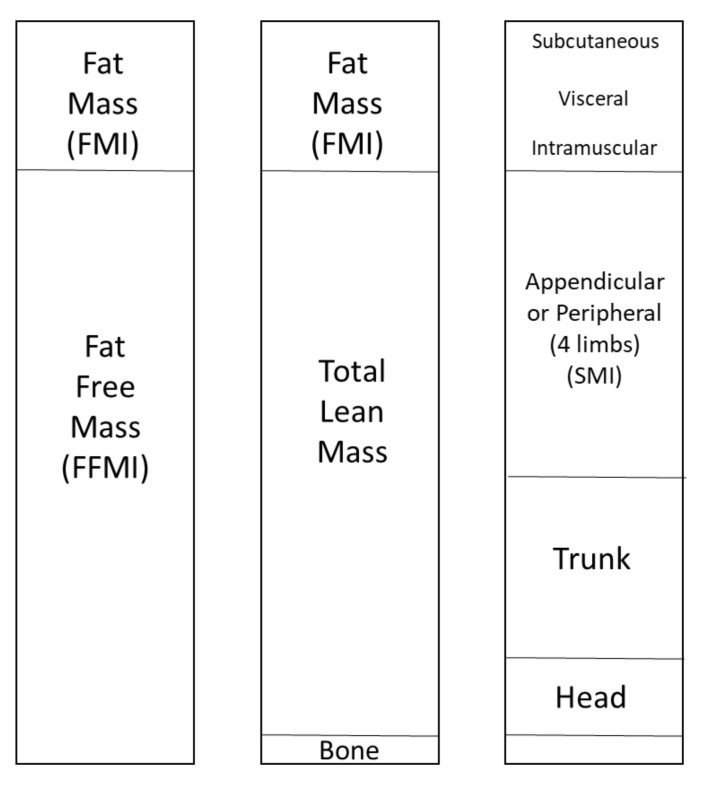
DXA compartment view of body composition.

The reference technique for measuring body composition is two-photon X-ray absorptiometry (DXA). DXA can assess the three primary compartments of body composition; namely, lean mass, fat mass, and bone mass [10] (Figure 1). DXA analyses have also been performed to evaluate subcutaneous adipose tissue (SAT) and visceral adipose tissue (VAT), which have different metabolic profiles [11]. The lower extremities can be assessed with peripheral quantitative computed tomography (pQCT), a non-invasive imaging technique. pQCT was used to measure muscle density and intramuscular adipose tissue, which histologically corresponds to the adipose tissue between fascicles [12]. From these measurements, the muscle surface area (i.e., cross-sectional area, CSA), the subcutaneous fat area (i.e., fat area), and intramuscular adipose tissue (IMAT) infiltration could be estimated [13]. A previous study in young girls demonstrated strong agreement between adipose tissue measured by pQCT and MRI for subcutaneous adipose tissue, skeletal muscle adipose tissue, and muscle density [13]. Visceral adipose tissue and ectopic fat accumulations in non-adipose tissues, such as liver, muscle, and heart, correspond to metabolically active fat, which is associated with insulin resistance and cardiovascular diseases [14,15,16]. Hence, the concept of adiposopathy has emerged, highlighting the importance of identifying the IMAT by new imaging techniques because of its consequences on metabolic health [17]. In contrast, SAT in the lower limbs might play a protective role by reducing the risk of cardiovascular disease and cancer. SAT might confer these beneficial effects by acting as a metabolic buffer for the influx of dietary lipids and by protecting other tissues from the lipotoxicity associated with excess lipids and ectopic fat deposits [17].

Muscle and fat assessments with pQCT were previously correlated with total body composition, based on DXA measurements, in the general population [18]. However, few studies have provided data for patients with RA [19,20], and no data are available for patients with SpA. In RA, altered muscle density, an indirect indicator of IMAT infiltration, was associated with impaired physical capacity, regardless of total adipose tissue distribution [19]. To our knowledge, no study has evaluated muscle quality in SpA.

The present pilot study aimed to analyze ectopic IMAT in patients with RA and SpA, based on pQCT measurements of IMAT and muscle density. Then, we examined correlations between ectopic IMAT and total body composition (measured with DXA), muscle function, and disease characteristics.

## 2. Materials and Methods

### 2.1. Study Population

Patients over 18 years old with active RA or SpA that visited the Rheumatology Department of Clermont-Ferrand University Hospital were consecutively invited to participate in the longitudinal RCVRIC cohort. The RCVRIC study aimed to analyze cardiovascular risk and chronic inflammatory rheumatism (PHRC RCVRIC AOI 2014 N° ID-RCB-A01847-40). The patients fulfilled the American College of Rheumatology (ACR)/European League Against Rheumatism (EULAR) 2010 criteria for RA [21] or ASAS criteria for axial [22] or peripheral SpA [23]. All patients were naïve of biologic drugs at baseline. Patients with RA could have received one or more conventional disease-modifying antirheumatic drugs (DMARDs), and patients with SpA could have received nonsteroidal anti-inflammatory drugs (NSAIDs) or conventional DMARDs. For the present study, we included only patients that had undergone both DXA and pQCT at baseline, between April 2014 and January 2018. The study was approved by the local ethics committee of Clermont-Ferrand (Institutional Review Boards: AU 1161). All patients were informed of the study and consented to participate.

### 2.2. Clinical and Imaging Data

We collected clinical and demographic data. We recorded the disease duration, the presence of rheumatoid factor and/or anti-CCP antibodies, and the detection of biological markers of inflammation, including the erythrocyte sedimentation rate (ESR; mm/h) and the circulating concentration of C-reactive protein (CRP; mg/L). Disease activity was evaluated with the disease activity score (DAS) 28 ESR/CRP for RA and with the Bath ankylosing spondylitis disease activity index (BASDAI) and ankylosing spondylitis disease activity score (ASDAS)-CRP for SpA. We recorded radiographic erosions detected on baseline images of feet and hands, for RA, and the presence of sacroiliitis on radiographs or MRIs, for SpA. Functional disability was evaluated with the health assessment questionnaire (HAQ), the Rheumatoid Arthritis Impact of Disease (RAID) score, and the Bath ankylosing spondylitis functional index (BASFI) score. We also recorded the use of conventional DMARDs, steroids, and NSAIDs.

BMI was calculated for all patients. Muscle strength was assessed by measuring the grip strength of the dominant hand (Handgrip test). Physical performance was assessed with a 6-minute walk test. Sedentary time was assessed on the GPAQ questionnaire, with the question: “How much time do you usually spend sitting or reclining on a typical day?”

### 2.3. Body Composition Assessments with DXA

A body composition analysis was carried out for each patient with DXA (HOLOGIC Discovery A S/N 85701). We estimated fat mass, lean mass, and bone mass of the whole body, and then, for specific regions of interest (e.g., appendages, trunk, and gynoid and android regions) using the manufacturer’s validated software (Version 4.02 HOLOGIC Apex). The percentage of body fat was calculated as the proportion of total body mass that consisted of fat mass. The fat mass index (FMI) was calculated as the total fat mass divided by height squared (kg/m^2^), and the fat free mass index (FFMI) was calculated as the total lean mass plus the bone mass divided by the height squared (kg/m^2^). The skeletal muscle mass index (SMI) was calculated as the appendicular lean mass divided by the height squared (kg/m^2^). The trunk-to-peripheral fat ratio, a measure of “android” fat, was calculated as the total fat of the body trunk divided by the fat in the periphery (legs and arms). The SAT and VAT were estimated by two blinded assessors with a validated method, by separating the SAT and VAT depots inside a region of interest with software recently developed for DXA [2].

### 2.4. Muscle and Fat Assessments with pQCT

Muscle and fat were measured with pQCT (XCT3000 Stratec Medizin technik GmbH, Pforzheim Germany) scans of the non-dominant leg. Scans were acquired at the tibia, at a site located at 66% of the height from the malleolus. The scanning speed was 40 mm/s, the voxel size was 0.5 mm, and the section thickness was 2.4 mm. All scans were analyzed with Stratec Software version 6.20. The measured parameters were: muscle density, fat area, and muscle area. The fat and muscle cross-sectional areas (CSAs, mm^2^) were obtained with edge-detection and threshold techniques, based on the attenuation characteristics, which are directly related to the density and composition of the tissues [19]. A threshold of −101 mg/cm^3^ was used to identify the skin; a threshold of 40 mg/cm^3^ was used to separate muscle and fat; and a threshold of 710 mg/cm^3^ was used to separate muscle and bone. The bone surface was identified at a threshold of 149 mg/cm^3^. Muscle quality was calculated as the strength-to-CSA ratio [24].

Muscle density was calculated mathematically from the scan data. In histopathology, the measured density was strongly correlated with the lipid content of muscle fibers [25]; thus, the higher the density, the lower the fatty infiltration [26]. Unfortunately, the algorithms proposed by the pQCT manufacturer did not allow direct measurements of IMAT (mm^2^); instead, the software estimated IMAT based on muscle density. Therefore, we used the algorithm of Schiferl, described by Blew et al. [27,28]. This procedure was written specifically for Stratec Software v 6.2; it uses image filtering and analysis modes to measure IMAT, based on points of density within muscle areas [29]. The advantage of this procedure was that it quantified the amount of patient movement during the examination; therefore, it estimated the quality of the acquisitions. Briefly, the algorithm developed by Schiferl and colleagues measured the percentage of movement (pmove) in the scanned area during acquisition. The acquisitions were considered reliable when the pmove was <25% [27].

### 2.5. Statistical Analysis

Data storage and management conformed to international guidelines. Continuous data are expressed as the mean and standard deviation or the median and interquartile range, according to the statistical distribution. Data normality was analyzed with the Shapiro–Wilk test. Relationships between pQCT and DXA data were analyzed with correlation coefficients: Pearson’s or Spearman’s, according to the statistical distribution of the variables. In addition to these analyses, mediation analysis was performed to assess the contribution of the lean mass to the relationship between the VAT and IMAT. Mediation analysis quantifies the extent to which a variable participates in the transmittance of change from a cause to its effect. It is a statistical method to test whether the effects of X (the independent variable) on Y (the dependent variable) operate through a third variable, M (the mediator). We estimated the mediation proportion by determining how much of the correlation between VAT and IMAT could be explained by the mediator (lean mass) [30,31]. Age and sex were considered potential confounders that were adjusted for in the mediation analysis. When variables did not follow a Gaussian distribution, a logarithmic transformation was applied. All statistical analyses were performed with Stata software (version 15, StataCorp, College Station, TX, USA). We assumed a two-sided type I error at 5%, and we applied a Sidak’s type I error correction to take into account multiple comparisons. The sample size was estimated to highlight relevant relationships between ectopic intramuscular fat (IMAT) or muscle density measured by pQCT and total body composition measured by DXA. For a correlation coefficient higher than 0.6, it was necessary to recruit at least 20 patients for each disease (RA and SpA) for a two-sided type I error at 5% and a statistical power greater than 80%.

## 3. Results

### 3.1. Patient Characteristics

Between 2014 and January 2018, 219 biologic-naïve patients were included in the cohort (89 with RA and 130 with SpA). Among these, the present study included 20 patients with RA and 23 patients with SpA that had undergone total body DXA and pQCTs. The main characteristics of these patients are presented in Table 1, and the corresponding pQCT and DXA results are presented in Table 2.

The patients were mainly women (75% of those with RA, 56.5% of those with SpA), with mean ages of 58.5 ± 14.2 years (RA) and 42.9 ± 14.2 years (SpA). The median disease durations were 4.2 ± 6.4 years for RA and 4.9 ± 8.0 years for SpA. Axial SpA was reported in 52.1% of patients with SpA. The mean disease activities were DAS28: 4.09 ± 1.36, BASDAI: 49.7 ± 17.6, and ASDAS-CRP: 3.1 ± 0.7. Methotrexate had been taken by 80% of patients with RA and 74% of patients with SpA, and steroids had been taken by 50% of patients with RA and 5% of patients with SpA. Patients with RA had weaker handgrip strength, higher BMIs, and higher body fat percentages than patients with SpA.

### 3.2. Correlations between pQCT and Total Body Composition (DXA)

The pQCT measurements were reliable; the average pmoves were 0.16 ± 0.13 for RA and 0.19 ± 0.35 for SpA.

In both RA and SpA, the IMAT was significantly correlated with BMI (r = 0.47 and 0.76, respectively; *p* < 0.05), the trunk-to-peripheral fat ratio (r = 0.4962 and 0.6957, respectively; *p* < 0.05), and VAT (r = 0.5143 and 0.6314, respectively; *p* < 0.05; Table 3 and Table 4). There were no correlations between IMAT and total fat mass, the percentage of fat, FMI, or SAT. In addition, in both RA and SpA, IMAT was significantly correlated with the DXA lean mass parameters, including total lean mass (r = 0.5414 and 0.8132, respectively; *p* < 0.05), SMI (r = 0.5825 and 0.8386, respectively; *p* < 0.05), and FFMI (r = 0.6877 and 0.8211, respectively; *p* < 0.05). In SpA only, IMAT was significantly correlated with hand muscle strength (r = 0.5316 *p* < 0.05), and CRP (r = 0.7219 *p* < 0.05). In RA, there was no significant correlation between IMAT and muscle function. Furthermore, IMAT was not correlated with other characteristics of the disease in either RA or SpA.

We also analyzed muscle density, another indirect method for assessing IMAT infiltration (i.e., the higher the fat infiltration, the lower the muscle density). Density was negatively correlated with VAT and the trunk-to-peripheral fat ratio in SpA (r = −0.5227 and r = −0.4269, respectively; *p* < 0.05) and with the lean body mass indices (Table 3 and Table 4). Density was also associated with the 6-minute walk test in RA (r = 0.6261 *p* < 0.05).

In contrast to IMAT, the fat area assessed with pQCT, in both RA and SpA, was significantly correlated with total fat mass (r = 0.6767 and 0.5089, respectively; *p* < 0.05), body fat percentage (r = 0.7068 and 0.6314, respectively; *p* < 0.05), and SAT (r = 0.6526 and 0.5524, respectively; *p* < 0.05). However, the fat area was not correlated with VAT. In RA, the fat area was inversely correlated with the 6-minute walk test (r = −0.8130 *p* < 0.05) and with handgrip strength (r = −0.5244 *p* < 0.05; Table 3). Moreover, in RA, we noted a significant correlation between the fat area and disease activity (DAS28 and DAS28CRP, respectively: r = 0.5239 and 0.4915, respectively; *p* < 0.05). In SpA, the fat area was significantly correlated with a worse handicap (HAQ, r = 0.6407, *p* < 0.05) and a worse 6-minute walk test (r = −0.5013 *p* < 0.05).

Although the CSA does not exclude fatty infiltration in muscle areas, in RA and SpA, the muscle CSA was significantly correlated with the indices of lean mass, including total lean mass (r = 0.5940 and 0.8597, respectively; *p* < 0.05), FFMI (r = 0.5534 and 0.8491, respectively; *p* < 0.05), and SMI (r = 0.4579 and 0.7509, respectively; *p* < 0.05), and also with the VAT (r = 0.4571 and 0.6393, respectively; *p* < 0.05). In SpA, the muscle CSA was also associated with BMI (r = 0.7509, *p* < 0.05). A significant association between muscle CSA and muscle strength was observed in both RA and SpA (r = 0.6839 and 0.5641, respectively; *p* < 0.05), and with disease impact in RA (RAID r = −0.5750, *p* < 0.05). In SpA alone, muscle CSA was correlated with CRP (r = 0.7528, *p* < 0.05). The strength-to-CSA ratio indicates the muscle strength per section of muscle, and thus, muscle quality. In RA, this ratio was negatively correlated with the RAID (r = −0.6923, *p* < 0.05) and with the sedentary time (r = −0.5529, *p* < 0.05) in SpA.

Because the sex differences in body composition could explain some differences between RA and SpA, we analyzed women separately (15 RA and 10 SpA) (Supplementary File S1). The significant correlation between IMAT and lean mass persisted both in RA and SpA women, whereas it persisted for BMI only in SpA and was not observed for VAT both in RA and SpA. A correlation between fat area and fat mass was noted both in RA and SpA women. In SpA women, the fat area was correlated with VAT, whereas in RA women it correlated with lean mass. CSA was correlated with BMI and lean mass in SpA women.

### 3.3. Mediation Analysis

**Figure 2:**
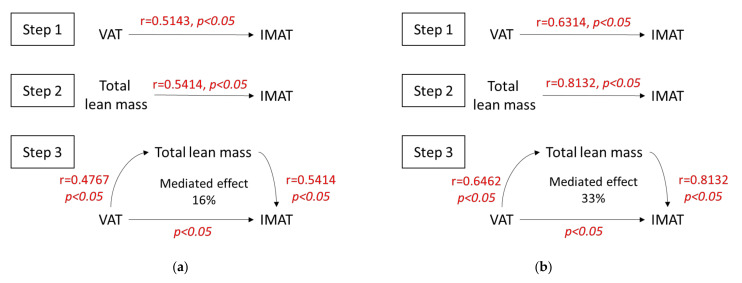
Mediation analysis of contributors to IMAT. Diagrams show three steps of the mediation analyses for (**a**) RA and (**b**) SpA. Tested mediator: total lean mass. Independent variable: VAT. Step 1: demonstration that VAT is correlated with IMAT. Step 2: demonstration that total lean mass is correlated with IMAT. Step 3: calculation of the influence of the mediator, total lean mass, on IMAT, after accounting for the direct effect of the independent variable, VAT.

In addition to the significant correlations shown above between the IMAT and VAT and between the IMAT and lean mass, we also observed that the total lean mass was significantly correlated with VAT in both RA and SpA (r = 0.4767, *p* < 0.05 and r = 0.6462, *p* < 0.05, respectively). Next, we performed a mediation analysis to determine the effect of the total lean mass on the correlation between the VAT and the IMAT (Figure 2).

We found that the VAT and the total lean mass values were significantly associated with the IMAT (steps 1 and 2 of the mediation analysis, respectively). In RA, these correlation coefficients were r = 0.5143 (*p* < 0.05) and r = 0.5414 (*p* < 0.05), respectively. In SpA, the correlation coefficients were r = 0.6314 (*p* < 0.05) and r = 0.8132 (*p* < 0.05), respectively.

We then tested the total lean mass as a mediator of the effect of VAT on IMAT. We found that the direct association between VAT and IMAT remained significant, and 16% and 33% of the effects of VAT on IMAT were mediated by the total lean mass in RA and SpA, respectively.

## 4. Discussion

We demonstrated that ectopic IMAT could be measured directly with pQCT in patients with active RA or SpA. We found that IMAT was associated with VAT and trunk fat, two metabolically active fat depots. In contrast, IMAT was not correlated with SAT or with total body fat, which were associated with fat area. We also observed that muscle density showed the same correlations. Indeed, muscle density is an indirect estimation of IMAT, and it was correlated with fatty infiltration in histological examinations [25]. These data suggest that metabolically active fat was specifically associated with IMAT in RA and SpA.

Given the statistically significant correlations between total lean mass and both IMAT and VAT, for the present study, we wondered whether total lean mass was a confounding factor in the association between VAT and IMAT. In previous studies, the IMAT (which corresponds to intramuscular ectopic fat) could not be isolated with DXA, and consequently, it was integrated into the total lean mass. Therefore, we hypothesized that, as the proportion of lean mass (measured with DXA) increased, the proportion of IMAT must also increase. In the present study, we performed a mediation analysis to assess the contribution of the total lean mass in mediating the relationship between VAT and IMAT. We demonstrated that lean mass mediated only 16% in RA and 33% in SpA of the correlation between VAT and IMAT. Thus, we concluded that most of the correlation between VAT and IMAT was independent of lean mass.

The association between IMAT and lean mass also suggested that measures of IMAT should be corrected by the appendicular lean mass of lower limbs. Histologically, IMAT constitutes a deposit of adipocytes between the muscle fascicles, and thus, it depends on the total muscle volume. Some high-risk conditions, such as ageing, obesity, hyperglycemia, and a sedentary lifestyle, might favor the differentiation of satellite cells into adipocytes, and thus, promote the accumulation of lipids [32]. In older populations, associations have been found between IMAT and impaired muscle function, balance disorders, and elevated CRP levels. However, although it has been suggested that the IMAT assessed with pQCT corresponded to the fat between groups of muscle fibers and fasciae, in practice, pQCT could not distinguish between intra- and inter-muscular fat [26,33]. Muscle density appears to be a better indicator of physical function and therefore of muscle quality, which may partly explain why it is inversely correlated with lean mass indices which more accurately reflect the quantity of muscle. In a previous study of 60 patients with RA [20], muscle density, but not IMAT, was associated with physical function (i.e., gait speed, quadriceps strength) and less disability. In our study, muscle density was associated with the 6-minute walk test in RA and less sedentary time in SpA, and was inversely correlated with lean body mass. IMAT was only associated with muscle strength in SpA. We also found that muscle area was correlated with handgrip strength and less severe disease (RAID), and that fat area was associated with disease activity (DAS28) and inversely correlated with the 6-minute walk test in RA. In older subjects, it was previously found that fatty infiltration increased with age and that the loss of strength was 2- to 5-fold greater than the loss of muscle mass, due to a loss of muscle quality with aging. That finding led to the proposal that the strength to-CSA ratio, which indicates muscle strength per section of muscle, could serve as a marker of muscle quality [15]. We found that this ratio was inversely correlated with disease severity (RAID), in RA, and a sedentary lifestyle in SpA.

Few studies have compared pQCT-based measurements of muscle and fat in the calf and DXA-based measurements of total body composition. A previous study in children without chronic diseases [34], and more recently, a study in healthy adults [18], showed that the fat parameters measured with pQCT were significantly associated with the total fat mass measured with DXA. Moreover, the muscle area measured with pQCT was also significantly associated with the total lean mass measured with DXA. In contrast, no association was found between muscle density and either muscle area or lean mass, in healthy adults [18]. Consistent with those results, we noted a good correlation between the muscle and fat areas measured with pQCT and the lean and fat masses assessed with DXA, in patients with chronic inflammatory rheumatic diseases. We correlated low muscular density and increased VAT, as previously reported in RA by Baker et al. [19].

The main strength of our work was the combined analysis of body composition with DXA and pQCT. Few studies have compared these two techniques. In addition, we used three indicators of muscle quality, based on pQCT measurements (IMAT, density, and the strength-to-CSA ratio) to best characterize ectopic intramuscular fat. Currently, the diagnostic performance of these measurements as a muscle quality assessment index remains to be established. Furthermore, our mediation analysis revealed that lean mass served as a mediator of the correlation between IMAT and VAT.

The main limitation of our study was the small sample size. The small group sizes may have limited our statistical power in detecting less marked correlations. Moreover, the control group matched for age and sex from the general population is lacking. The objective of the study was not to compare RA and SpA patients, therefore different by definition in terms of sex and age, but to study the relationship between body composition variables (measured by pQCT and DXA) in representative samples of the RA and SpA populations. Body composition differs between men and women and could contribute to explain the differences observed between RA and SpA patients, in particular for BMI, body fat but also for ectopic fat deposits (Table 3). Women have higher fat mass, and lower lean mass compared to men [35]. However, men have higher VAT and myosteatosis associated with a higher cardiometabolic risk compared to women. In contrast, higher lower limb fat was associated with a more favorable cardio metabolic profile in women compared to men [35]. In addition, in this cross-sectional study, only associations can be demonstrated. Thus, prospective studies will be necessary in order to analyze the evolution with DMARD treatment or after additional intervention such as nutrition or physical exercise.

## 5. Conclusions

This pilot study showed that ectopic intramuscular fat, assessed with pQCT in the calf, was correlated with VAT, but not whole body fat assessed with DXA, in patients with chronic inflammatory rheumatic diseases. This finding suggests that IMAT was specifically associated with metabolically active fat. The association between IMAT and lean mass also suggests that measures of IMAT should be corrected for the appendicular lean mass of the lower limbs. We demonstrated that muscle and fat areas measured with pQCT were correlated with lean and fat masses assessed with DXA, respectively, and with physical function and disability. However, the best index for assessing muscle quality remains to be established.

## Figures and Tables

**Table 1:** Characteristics of patients with rheumatoid arthritis (RA) or spondyloarthritis (SpA).

Characteristic	RA *n* = 20	SpA *n* = 23
Age (years)	58.8 ± 14,2	42.9 ± 11.1
Sex (female)	15 (75)	13 (56.5)
Disease duration (years)	4.2 ± 6.4	4.9 ± 8.0
BMI (kg/m^2^)	28.6 ± 7.1	25.3 ± 4.6
Rheumatoid factor	14 (82.3)	NA
Anti-CCP	13 (76.4)	NA
Axial SpA	NA	12 (52.1)
Peripheral SpA	NA	9 (39.1)
Radiographic sacroiliitis	NA	6 (26.0)
MRI sacroiliitis	NA	7 (30.4)
HLA B27	NA	15 (65.2)
DAS28	4.09 ± 1.36	NA
DAS28 CRP	3.79 ± 1.16	NA
BASDAI	NA	49.7 ± 17.6
BASFI	NA	41.9 ± 22.6
ASDAS-CRP	NA	3.1 ± 0.7
RAID	5.97 ± 1.74	NA
HAQ	0.897 ± 0.518	0.664 ± 0.314
VS (mm/h)	24.6 ± 17.9	18.2 ± 14.4
CRP (mg/L)	22.3 ± 29.6	15.3 ± 19.0
NSAID current use	3 (16.6)	14 (70)
Corticosteroids current use	10 (50)	1 (4.7)
Methotrexate current use	16 (80)	17 (73.9)
Muscle strength (handgrip; kg)	16.12 ± 14.29	28.73 ± 15.66
6-minute walk test (m)	423 ± 101	457 ± 104
Sedentary time (GPAQ_Q16) (minutes/week)	2618 ± 1733	2142 ± 1528

Data are presented as frequencies (associated percentages) or as mean ± standard deviation, as indicated. BMI: body mass index; CCP: cyclic citrullinated peptide; MRI: magnetic resonance imaging; DAS28: disease activity score in 28 joints; CRP: C-reactive protein; BASDAI: Bath ankylosing spondylitis disease activity index; BASFI: Bath ankylosing spondylitis functional index; ASDAS: ankylosing spondylitis disease activity score; RAID: rheumatoid arthritis impact of disease; HAQ: health assessment questionnaire; ESR: erythrocyte sedimentation rate; NSAIDs: nonsteroidal anti-inflammatory drugs.

**Table 2:** Body composition parameters measured with peripheral quantitative computed tomography and two-photon X-ray absorptiometry in patients with RA or SpA.

Parameter	RA *n* = 20	SpA *n* = 23
Muscle area (CSA; mm^2^)	6166 ± 1541	7031 ± 1322
Fat area (mm^2^)	3672 ± 2093	2402 ± 1297
IMAT (mm^2^)	1488 ± 556	1760 ± 537
Muscle density (mg/cm^3^)	77.01 ± 2.94	77.16 ± 3.08
Strength-CSA ratio (kg/mm^2^)	0.0024 ± 0.0017	0.0039 ± 0.0019
BMI (kg/m^2^)	28.6 ± 7.1	25.3 ± 4.6
Total fat mass (kg)	25.13 ± 12.34	20.94 ± 7.82
Fat percentage	31.57 ± 9.54	27.22 ± 7.21
FMI (kg/m^2^)	10.35 ± 5.75	7.23 ± 2.87
Visceral adipose tissue (cm^2^)	83.58 ± 61.22	86.19 ± 66.46
Subcutaneous adipose tissue (cm^2^)	332.3 ± 174.8	262.6 ± 121.7
Trunk–peripheral fat ratio	0.8394 ± 0.2931	0.9767 ± 0.2936
Total lean mass (kg)	52.57 ± 16.43	55.99 ± 15.68
FFMI (kg/m^2^)	23.04 ± 18.56	18.97 ± 3.91
SMI (kg/m^2^)	8.26 ± 2.07	8.03 ± 1.55

Data are presented as frequencies (associated percentages) or as the mean ± standard deviation, as indicated. IMAT: intramuscular adipose tissue; CSA: cross-sectional area; FMI: fat mass index; FFMI: fat-free mass index; SMI: skeletal muscle mass index.

**Table 3:** Associations in RA between muscle parameters measured with pQCT, total body composition parameters measured with DXA, muscle function, and disease characteristics.

Parameter	Muscle Area (CSA, mm^2^)	Fat Area (mm^2^)	IMAT (mm^2^)	Muscle Density (mg/cm^3^)	Strength-to-CSA Ratio (kg/mm^2^)
BMI (kg/m^2^)	0.2632	0.4923 *	0.4757 *	−0.4779	0.0214
Total fat mass (kg)	0.0496	0.6767 *	0.3053	−0.3474	−0.0089
Fat percentage	−0.3053	0.7068 *	−0.0932	−0.0737	−0.2147
FMI (kg/m^2^)	0.0165	0.6526 *	0.2556	−0.2579	−0.1403
Visceral adipose tissue (cm^2^)	0.4571 *	0.0932	0.5143 *	−0.2789	0.2201
Subcutaneous adipose tissue (cm^2^)	0.1113	0.6526 *	0.3053	−0.2965	−0.0857
Trunk-to-peripheral fat ratio	0.3474	0.0511	0.4962 *	−0.2684	−0.0294
Total lean mass (kg)	0.5940 *	0.0286	0.5414 *	−0.3263	0.3826
FFMI (kg/m^2^)	0.5534 *	0.2436	0.6827 *	−0.4491	0.1477
SMI (kg/m^2^)	0.4579 *	0.2123	0.5825 *	−0.5562 *	0.1901
Muscle strength (handgrip test; kg)	0.6839 *	−0.5244 *	0.1713	0.2704	0.9926 *
6-minute walk test (m)	0.6539 *	−0.8130 *	0.1635	0.6261 *	0.5022
Sedentary time (GPAQ_Q16) (minutes/week)	0.3002	−0.3671	0.0597	0.4324	0.0308
DAS28	−0.3642	0.5239 *	−0.1466	−0.1074	−0.4200
DAS28CRP	−0.2767	0.4915 *	−0.0248	0.0331	−0.3718
CRP (mg/L)	−0.0565	0.2599	0.1469	−0.3938	−0.4720
HAQ	−0.2952	0.3399	0.0698	−0.3564	−0.5152
RAID	−0.5750 *	0.2250	−0.2786	−0.1604	−0.6923 *

Values are Spearman correlation coefficients; * *p* < 0.05. BMI: body mass index; FMI: Fat mass index; FFMI: Fat-free mass index; SMI: Skeletal muscle mass index; DAS28: disease activity score in 28 joints; CRP: C-reactive protein; HAQ: health assessment questionnaire; RAID: rheumatoid ar-thritis impact of disease.

**Table 4:** Associations in SpA between muscle parameters measured with pQCT, body composition parameters measured with DXA, muscle function, and disease characteristics.

Parameter	Muscle Area (CSA, mm^2^)	Fat Area (mm^2^)	IMAT (mm^2^)	Muscle density (mg/cm^3^)	Strength-to-CSA Ratio (kg/mm^2^)
BMI (kg/m^2^)	0.7509 *	0.0158	0.7632 *	−0.3632	0.2588
Total fat mass (kg)	0.3004	0.5089 *	0.4051	−0.3696	0.2772
Fat percentage	−0.2974	0.6314 *	−0.2668	−0.0069	−0.3474
FMI (kg/m^2^)	0.1443	−0.0958	0.2678	−0.3043	−0.1035
Visceral adipose tissue (cm^2^)	0.6393 *	0.0049	0.6314 *	−0.5227 *	0.5544 *
Subcutaneous adipose tissue (cm^2^)	0.2747	0.5524 *	0.3794	−0.3577	0.1982
Trunk-to-peripheral fat ratio	0.7688 *	−0.1868	0.6957 *	−0.4269 *	0.5368 *
Total lean mass (kg)	0.8597 *	−0.1621	0.8132 *	−0.4249 *	0.6140 *
FFMI (kg/m^2^)	0.8491 *	−0.1868	0.8211 *	−0.4269 *	0.3404
SMI (kg/m^2^)	0.7509 *	−0.3281	0.8386 *	−0.3474	0.3824
Muscle strength (handgrip test; kg)	0.5641 *	−0.2329	0.5316*	−0.2750	0.9227 *
6-minute walk test (m)	0.1182	−0.5013 *	−0.0602	0.1889	0.1993
Sedentary time (GPAQ_Q16) (minutes/week)	0.1471	−0.0940	0.1524	0.1228	−0.5529 *
BASDAI	−0.2656	0.2786	−0.1690	−0.0277	−0.4072
BASFI	−0.2852	0.3741	−0.1462	−0.3215	−0.3431
ASDAS CRP	0.1163	0.2327	0.4183	−0.4050	−0.3586
CRP (mg/l)	0.7528 *	−0.1104	0.7219 *	−0.1366	0.1687

Values are Spearman correlation coefficients; * *p* < 0.05. BMI: body mass index; FMI: Fat mass index; FFMI: Fat-free mass index; SMI: Skeletal muscle mass index; BASDAI: Bath ankylosing spondylitis disease activity index; BASFI: Bath ankylosing spondylitis functional index; ASDAS: ankylosing spondylitis disease activity score; CRP: C-reactive protein.

## Data Availability

The data presented in this study are available on request from the corresponding author.

## Supplementary Materials

The following are available online at https://www.mdpi.com/article/10.3390/nu13124533/s1, File S1.

## Abbreviations

IMAT	Intramuscular adipose tissue
CSA	Cross-sectional area
FMI	Fat mass index
FFMI	Fat-free mass index
SMI	Skeletal muscle mass index
VAT	Visceral adipose tissue
SAT	Subcutaneous adipose tissue (cm^2^)
