# Supplementary material for: Assessment of Intramuscular Fat and Correlation with Body Composition in Patients with Rheumatoid Arthritis and Spondyloarthritis: A Pilot Study
Source: Nutrients. 2021 Dec 17;13(12):4533. doi: 10.3390/nu13124533 (PMC8707224; doi:10.3390/nu13124533)
Supplement: Supplementary file 1 [file nutrients-13-04533-s001.zip › nutrients-1448792-supplementary.pdf]

Associations in between muscle parameters measured with pQCT, and body composition parameters measured with DXA, in women.

In the 15 RA women, the IMAT was significantly correlated with total lean mass (r=0.5964, p <0.05). Muscle density was significantly correlated with the 6-minute walk test (r=0.7352, p <0.05), sedentary time (r=0.7256, p <0.05), and inversely with CRP level (r=-0.6579, p <0.05). The fat area was correlated with BMI (r=0.8036, p <0.05), SAT (r=0.7357, p <0.05), total fat mass (r=0.775 p <0.05), FMI (r=0.7821, p <0.05), total lean mass (r=0.7286, p <0.05), FFMI (r=0.7107, p <0.05), SMI (r=0.6835, p <0.05). CSA was correlated with handgrip strength (r=0.6397, p <0.05) and with the strength-to-CSA ratio (r=0.5944, p <0.05).

In the 13 SpA women, the IMAT was significantly correlated with BMI (r=0.7500, p <0.05), FMI (r=0.7364, p <0.05) and SMI (r=0.7333, p <0.05). No significant correlation was observed for muscle density. The fat area was correlated with BMI (r=0.7333, p <0.05), percentage of fat (r=0.8727, p <0.05), total fat mass (r=0.9091, p <0.05), FMI (r=0.8273, p <0.05), the trunk-to-peripheral fat ratio (r=0.6909, p <0.05), SAT (r=0.9182, p <0.05) and VAT (r=0.7273, p <0.05). CSA was correlated with BMI (r=0.6667, p <0.05), total lean mass (r=0.6364, p <0.05), FFMI (r=0.7091, p <0.05), and SMI (r=0.8667, p <0.05).
